# A case of severe visual loss related to treatment with pembrolizumab for metastatic renal pelvic cancer

**DOI:** 10.1002/iju5.12784

**Published:** 2024-09-06

**Authors:** Naoki Inoue, Mihoko Iitzuka, Hiroki Tanaka, Yudai Ishikawa, Naoko Kawamura, Tetsuo Okuno

**Affiliations:** ^1^ Department of Urology JA Toride Sogo Iryo Center Toride Japan; ^2^ Department of Ophthalmology JA Toride Sogo Iryo Center Toride Japan; ^3^ Department of Urology Tokyo Medical and Dental University Tokyo Japan

**Keywords:** immune‐related adverse events, optic neuritis, pembrolizumab, urothelial carcinoma, vision loss

## Abstract

**Introduction:**

Pembrolizumab is the standard therapy for urothelial carcinoma treatment; however, adverse events have been noted. Here, we report a rare case of vision loss as an immune‐related adverse event of pembrolizumab therapy in a patient with metastatic renal pelvic cancer.

**Case presentation:**

A 69‐year‐old man treated with pembrolizumab for lung and lymph node metastases of renal pelvic cancer experienced significant vision loss in both eyes after 11 treatment cycles. Without magnetic resonance imaging confirmation owing to an MRI‐unsafe pacemaker, his clinical features suggested immune checkpoint inhibitor‐associated optic neuritis. Pembrolizumab was discontinued, and the patient received steroid pulse and immunoglobulin therapy. His vision in the right eye improved, but that in the left eye remained unchanged. He maintained a partial response for 36 months despite pembrolizumab discontinuation.

**Conclusion:**

Despite its rarity, vision loss is a potential irAE in patients treated with ICIs, including pembrolizumab.

**Abbreviations & Acronyms**
CT: computed tomography
GC: gemicitabine and cisplatin
ICI: immune checkpoint inhibitor
irAE: immune‐related adverse event
MRI: magnetic resonance imaging
OCT: optical coherence tomography
PD: progressive disease
PD‐1: programmed death‐1
PR: partial response
US: ultrasonography

**Keynote message**

Vision loss during pembrolizumab treatment is rare, but should be carefully monitored. Herein, we present a case of optic neuritis related to the use of pembrolizumab in treating metastatic urothelial carcinoma.

## Introduction

Pembrolizumab is administered to patients with various malignancies, including advanced urothelial carcinoma. Reports of pembrolizumab‐associated irAEs have increased with its increasing use. Here, we report a case of vision loss associated with pembrolizumab administration in a patient with metastatic renal pelvic carcinoma.

## Case report

**Fig. 1:**
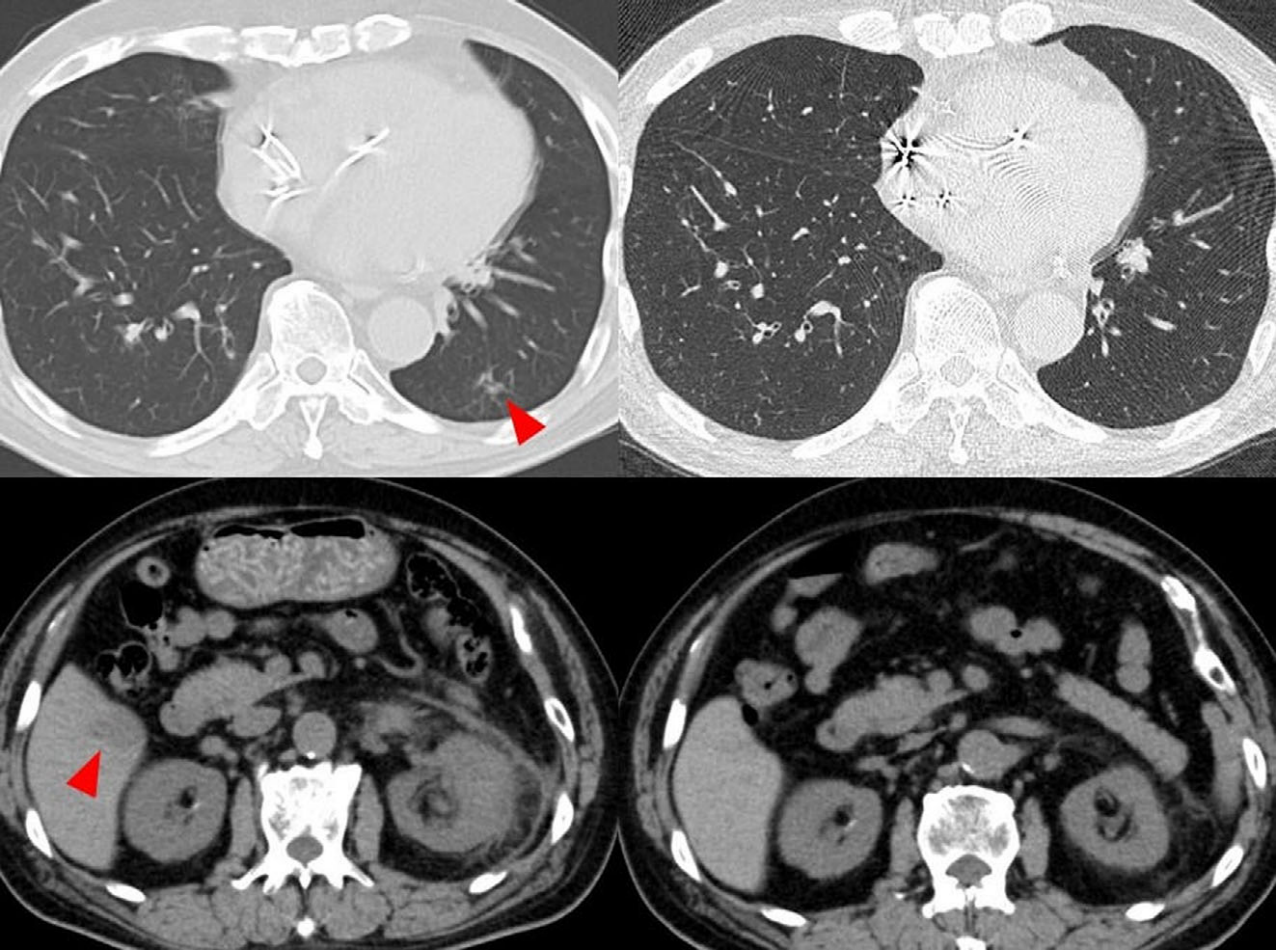
Before pembrolizumab treatment, lung metastases, liver metastases, and para‐aortic lymph node metastases were observed. After pembrolizumab administration, as shown in the figure, the lung and liver metastases disappeared. Similarly, the lymph node decreased in size.

A 69‐year‐old man presented with gross hematuria, and his urine cytology was classified as class IV. US and cystoscopy did not reveal bladder tumors; however, bleeding from the left ureteral orifice was detected. Contrast‐enhanced CT examination revealed a gradually enhancing tumor in the left kidney, a swelling para‐aortic lymph node, and lung metastases. An US‐guided renal biopsy was performed, and the pathological examination revealed urothelial carcinoma. Therefore, systemic chemotherapy with GC was initiated. Liver metastases appeared during the first course of GC therapy and persisted for six courses of GC treatment. CT for the evaluation of chemotherapy showed shrinkage of the primary lesion, liver, and para‐aortic lymph node metastases and growth of the lung metastases. In addition, blood tests showed that Grade 4 platelet count decreased (24 × 10^9^/L). PD was diagnosed, and pembrolizumab was started as second‐line therapy (200 mg/body administered every 3 weeks). Imaging evaluation at the end of two courses of pembrolizumab indicated a good response and resolution of the liver lesions (Fig. 1).

**Fig. 2:**
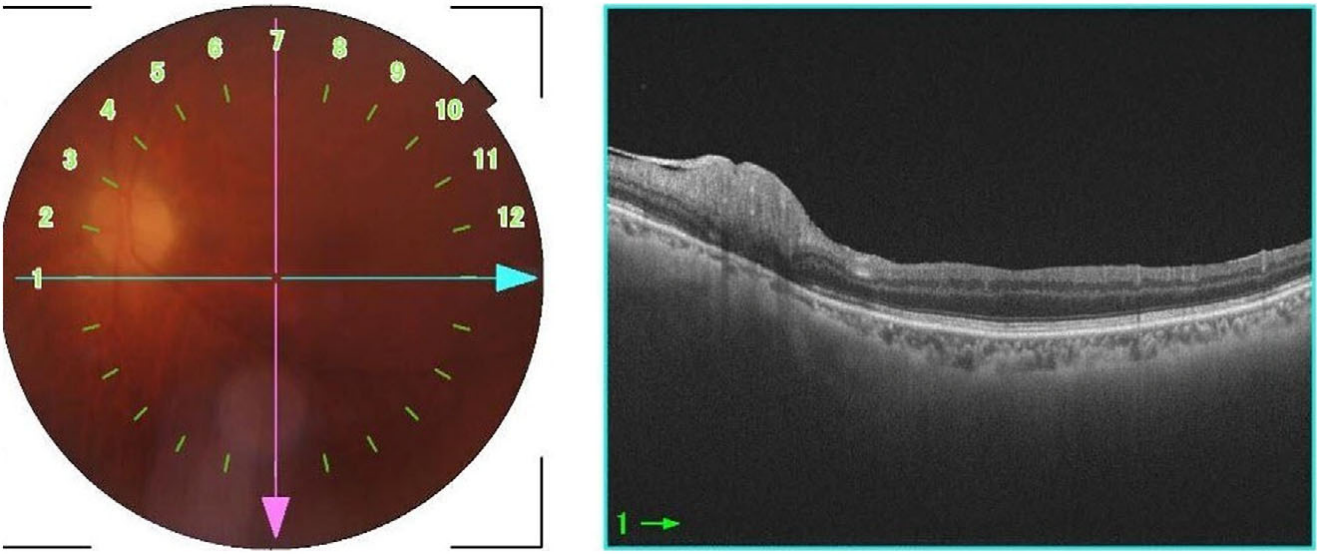
Initial fundus examination and OCT findings of the left eye. Fundus imaging showed some haziness and raised suspicion of optic disc swelling. OCT confirmed the presence of edema in the layer of nerve fibers.

**Fig. 3:**
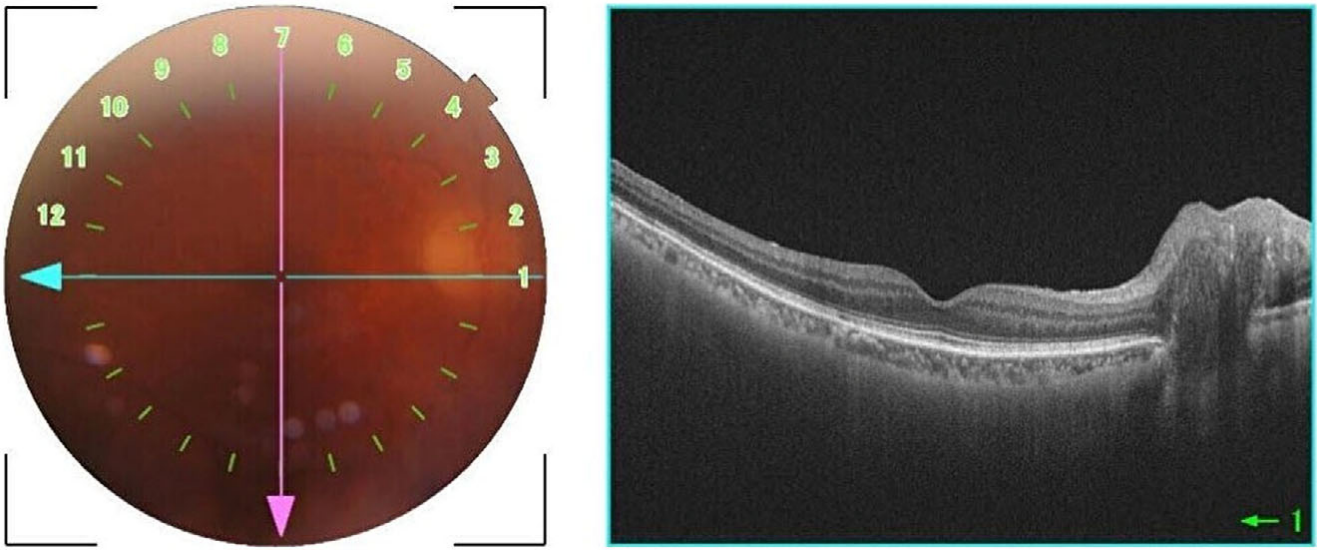
Fundus imaging and OCT of the right eye showed similar findings, including haziness and suspected optic disc swelling.

However, after 11 courses of pembrolizumab, vision loss occurred in both eyes. An ophthalmologic evaluation revealed a visual acuity of 0.5 in the right eye, no light perception in the left eye, and swelling of the optic discs, indicating optic neuritis in both eyes (Figs 2 and 3). Because the patient had an incompatible pacemaker, a head MRI examination could not be performed. Autoantibody tests ruled out autoimmune diseases, such as sarcoidosis, systemic lupus erythematosus, and Sjögren's syndrome.

ICI‐associated optic neuritis was suspected as pembrolizumab‐induced retrobulbar optic neuritis; therefore, pembrolizumab was discontinued, and steroid pulse therapy was started 6 days after the onset of vision loss, followed by immunoglobulin therapy. The right eye visual acuity improved within a few days, but that of the left eye remained unchanged. Pembrolizumab was not reinstituted, but the urothelial carcinoma had not progressed, and a PR was maintained for 36 months.

## Discussion

The KEYNOTE‐045 trial is a clinical trial for exploring pembrolizumab as a second‐line therapy for platinum‐refractory advanced urothelial carcinoma or metastatic urothelial carcinoma.^1^ This study showed that neurological disorders of all grades were present in 6.4% of patients, and ocular toxicity and optic neuritis were reported in few cases only. Previous reports have indicated that ocular toxicities are rare,^2^ accounting for less than 1% of all toxicities. ICIs, including pembrolizumab, cause ocular toxicities, such as dry eye, ocular myasthenia, uveitis, and eye inflammation. Neuro‐ophthalmic complications of ICIs occur in 0.46% of patients undergoing ICI treatment. In a previous study, the median time to eye symptom onset was two cycles, and pembrolizumab was the most common causative agent for neuro‐ophthalmic complications, followed by nivolumab.^3^

While common optic neuritis presents with the classic triad of unilateral visual disturbances, color vision impairment, and pain, ICI‐associated optic neuritis is characterized by a relatively high rate of bilateral visual disturbances (64%) and color blindness (67%). Clinical symptoms and abnormal optic nerve enhancement on MRI are useful for a definitive diagnosis of ICI‐associated optic neuritis.^4^

The present case suffered from bilateral painless visual loss but no color vision impairment, consistent with the clinical features of ICI‐associated optic neuritis. However, the patient had an MRI‐incompatible pacemaker; therefore, a head MRI examination could not be performed. Fundus examination revealed no abnormalities other than swelling of the optic nerve papillae, and autoantibody tests ruled out other autoimmune diseases. An association between vision loss and pembrolizumab was identified, and steroid pulses and immunoglobulin therapy were initiated, after which vision loss was partially reversed.

Treatment recommendations vary depending on the severity of irAEs. For grade‐1 and grade‐2 irAEs, oral corticosteroids at a dose of 0.5–1 mg/kg/day are generally administered, whereas for grade‐3 and grade‐4 irAEs, high‐dose oral corticosteroids (1–2 mg/kg/day) or steroid pulse therapy are typically administered.^5^ Systemic corticosteroids should be considered, as they can contribute to favorable visual recovery outcomes. Corticosteroid therapy is the primary treatment for the management of optic neuritis, augmented by plasmapheresis and intravenous immunoglobulins in select cases. Hahn and Pepple^6^ reported a case with neuroretinitis that involved resolving of the optic neuritis with topical and systemic corticosteroids. Early detection and diagnosis of optic neuritis can contribute to improved treatment outcomes.^7^ However, in the present case, despite steroid therapy, blindness was not alleviated in one of the eyes.

## Conclusion

Pembrolizumab, a PD‐1 inhibitor, has proven effective in treating various malignancies. Physicians must carefully consider visual events for early recognition and prompt treatment of visual dysfunctions. Effective collaboration among clinical departments is essential for the timely diagnosis and appropriate management of irAEs, leading to improved patient care and overall treatment success.

## Author contributions

Naoki Inoue: Conceptualization; formal analysis; writing – original draft; writing – review and editing. Mihoko Iitzuka: Writing – review and editing. Hiroki Tanaka: Writing – review and editing. Yudai Ishikawa: Writing – review and editing. Naoko Kawamura: Writing – review and editing. Tetsuo Okuno: Supervision; writing – review and editing.

## Conflict of interest

The authors declare no conflict of interest.

## Approval of the research protocol by an Institutional Reviewer Board

Not applicable.

## Informed consent

Informed consent was obtained from the participants in the study.

## Registry and the Registration No. of the study/trial

Not applicable.
